# Agronomic Factors Influencing the Scale of *Fusarium* Mycotoxin Contamination of Oats

**DOI:** 10.3390/jof7110965

**Published:** 2021-11-12

**Authors:** Oluwatobi Kolawole, Karl De Ruyck, Brett Greer, Julie Meneely, Fiona Doohan, Martin Danaher, Christopher Elliott

**Affiliations:** 1 Institute for Global Food Security, Queen’s University Belfast, Belfast BT9 5DL, UK; brett.greer@qub.ac.uk (B.G.); j.p.meneely@qub.ac.uk (J.M.); chris.elliott@qub.ac.uk (C.E.); 2 Teagasc Food Research Centre, Ashtown, D15 KN3K Dublin, Ireland; karlderuyck@pm.me (K.D.R.); martin.danaher@teagasc.ie (M.D.); 3 School of Biology and Environmental Science, College of Life Sciences, UCD, Belfield, D04 V1W8 Dublin, Ireland; fiona.doohan@ucd.ie

**Keywords:** mycotoxins, agricultural practices, mycotoxigenic fungi, *Fusarium*, oats, cereals, statistical analysis, agronomy

## Abstract

Seven agronomic factors (crop season, farming system, harvest date, moisture, county, oat variety, and previous crop) were recorded for 202 oat crops grown across Ireland, and samples were analysed by LC-MS/MS for four major *Fusarium* mycotoxins: deoxynivalenol (DON), zearalenone (ZEN), T-2 toxin and HT-2 toxin. Type A trichothecenes were present in 62% of crops, with 7.4% exceeding European regulatory limits. DON (6.4%) and ZEN (9.9%) occurrences were relatively infrequent, though one and three samples were measured over their set limits, respectively. Overall, the type of farming system and the previous crop were the main factors identified as significantly influencing mycotoxin prevalence or concentration. Particularly, the adherence to an organic farming system and growing oats after a previous crop of grass were found to decrease contamination by type A trichothecenes. These are important findings and may provide valuable insights for many other types of cereal crops as Europe moves towards a much greater organic-based food system.

## 1. Introduction

Oats (*Avena sativa*) are a whole-grain cereal crop belonging to the grass family (Poaceae) [1]. They are a very good source of carbohydrates and fibre (especially beta glucan), and are high in minerals, unsaturated fatty acids, vitamins, and quality protein with a good amino acid balance [1]. Due to their numerous benefits, including the lowering of blood sugar and cholesterol levels, oats and oat products have gained considerable attention as a health food [1,2]. Moreover, oats are suitable for inclusion in a gluten-free diet as the primary protein in oats is avenalin, compared with gluten in wheat and barley crops [1,2]. Diverse food products that can be produced from oats include porridge, beta-glucans, flapjacks, fat replacers, oat milk, oat oil, yoghurt drinks, protein powder, furfural, and animal feed [2,3]. 

However, oat crops are highly susceptible to *Fusarium* infection pre-harvest, particularly during the flowering stage. This leads to diseases such as *Fusarium* head blight, which drastically reduces oat crop yield and quality [4,5]. The most important *Fusarium* species in oats include *F. graminearum*, *F. poae*, *F. langsethiae*, *F. avenaceum*, *F. sporotrichioides*, and *F. culmorum* [5]. Aside from losses in yield and quality due to *Fusarium* infection, the accumulation of toxic *Fusarium* secondary metabolites, including deoxynivalenol (DON), zearalenone (ZEN), T-2 and HT-2 toxins, is also a major concern in oat production [6]. These toxic secondary metabolites (known as mycotoxins) are hazardous to human and animal health [7,8]. The exposure to DON, the most common of the type B trichothecenes, produces acute gastrointestinal symptoms in humans as well as the suppression of weight gain and anorexia in livestock animals [8]. Various adverse health effects, including immunotoxicity, hepatotoxicity, and infertility, were linked to the consumption of food and feed contaminated with ZEN [9]. T-2 and HT-2 toxins are closely related compounds with equivalent toxicities, and are representative species of type A trichothecenes [10]. The long-term exposure to these toxins inhibits protein, DNA and ribonucleic acid (RNA) synthesis [10]. Both compounds can also cause apoptosis, necrosis and haematotoxic effects, with a reduced production of leucocytes, erythrocytes, and platelets [10].

To safeguard human and animal health, the European Food Safety Authority (EFSA) established maximum limits for DON and ZEN in cereals and cereal products, while an indicative level was set for the sum of the T-2 and HT-2 toxins in cereals intended for human consumption (Commission Regulation (EC) No. 1881/2006) (Table 1) [11,12]. Following an evaluation of the occurrence and toxicity associated with chronic dietary exposure to DON, ZEN, and the sum of T-2 and HT-2, the EFSA established tolerable daily intakes (TDI) of 1, 0.25, and 0.02 μg/kg body weight (bw) per day, respectively [10,12,13].

In Ireland, according to the central statistics office (CSO), the land area used for the cultivation of either spring or winter oats is between 18,000 and 24,000 ha (~44,000–60,000 acres), with a yield of approximately 205,000 tonnes [14]. Previous surveys on mycotoxin incidence and the contamination levels in oats grown in Ireland showed that *Fusarium* mycotoxins, particularly T-2 and HT-2, frequently occur in oats at levels well above the regulatory limits [15,16,17]. Furthermore, the severity of pre-harvest infection and the propagation of toxigenic fungi within grain is highly dependent upon weather conditions; thus, mycotoxin concentrations observed at harvest vary widely from year to year [15,16,17]. Reports from Northern and Western European countries including the United Kingdom, Finland, Poland, and Sweden also showed that the composition of the *Fusarium* pathogen complex occurring on cereal heads, as well as mycotoxin types and concentrations, are variable and influenced by different agroclimatic factors [18,19,20,21,22,23,24].

In order to minimise the risk of exceeding the legal limits for *Fusarium* mycotoxins in cereals and cereal-based products, several organisations including the Food Standards Agency [25], Food and Agriculture Organisation (FAO) [26], and EFSA [27] published recommended measures based on good agricultural practices (GAP) such as crop rotation, irrigation, tillage, and the use of resistant varieties, altogether comprising a pre-harvest strategy to reduce mycotoxins in cereals and cereal-based products. As no previous studies examined the impact of different agricultural practices on the mycotoxin content of oats grown in Ireland, this study was carried out to evaluate and identify specific agronomic practices to ensure the reduction in major *Fusarium* mycotoxins, represented by DON, ZEN, T-2, and HT-2 in Irish oats.

## 2. Materials and Methods

### 2.1. Chemicals and Materials

Mycotoxin standards—DON, ZEN, T-2, and HT-2 toxins—were obtained from Romer Lab (Cheshire, UK). LC-MS grade methanol (MeOH) and acetonitrile (MeCN) were purchased from Honeywell (London, UK). Ultrapure water (resistivity of 18.2 MΩ-cm) was obtained from a Milli-Q system (Millipore, Burlington, MA, USA). LC-MS grade formic acid and syringe filters (PTFE, 0.22 μm pore size) were supplied by Sigma-Aldrich (St. Louis, MO, USA) and Waters (Wilmslow, UK), respectively.

### 2.2. Preparation of Standard Solutions

The stock solutions of DON, ZEN, T-2, and HT-2 were individually prepared by accurately weighing 5 mg of each mycotoxin and dissolving in 5 mL of acetonitrile, to obtain 1 mg mL^−1^ of each standard. Working standard solutions were prepared by diluting appropriate volumes of each stock standard solution with acetonitrile. All standard solutions were kept in amber glass vials at −20 °C and brought to room temperature before use. The stock solutions were renewed every 2 months, while multi-mycotoxin working solutions were prepared weekly.

### 2.3. Sampling

A total of 202 oats samples were collected from farmers and oat processors across Ireland between July and September 2020. These samples were analysed for the occurrence of DON, ZEN, T-2, and HT-2 toxins, using state-of-the art mass spectrometric instruments and techniques. Furthermore, agronomic data including crop varieties, fungicide application, storage conditions and duration, and farming practices, were collected. 

### 2.4. Sample Preparation

Briefly, 1.0 g ± 0.05 g of homogenised sample was weighed into a 50 mL polypropylene tube. Thereafter, 10 mL of acetonitrile:water:formic acid (79: 20: 1, *v*/*v*/*v*) was added, and vortexed for 45 min. Magnesium sulphate (1 g) and sodium chloride (0.2 g) were added, and the tube was immediately shaken for 30 s. After centrifugation at 4000× *g* for 10 min (15 °C) to induce separation of the aqueous phase from the organic phase, an aliquot of 1 mL of the upper organic phase was collected and filtered through a 0.2 µm PTFE syringe filter into an LC-MS/MS vial.

### 2.5. UHPLC-MS/MS Parameters

Qualitative and quantitative analyses of the target mycotoxins in oat samples were carried out on an ExionLC™ AD ultra-high-performance liquid chromatography system (Framingham, MA, USA) coupled to a SCIEX 5500+ QTrap triple quadrupole mass spectrometer (MS/MS) equipped with a Turbo V™ electrospray ionisation source (SCIEX, Framingham, MA, USA). Chromatographic separation was achieved using a Synergi 4 µm Fusion-RP 80 Å, 50 × 2 mm LC column, with the column maintained at 40 °C. The mobile phase was composed of water (A) and methanol:acetonitrile 50:50 (*v*/*v*) (B), both containing 1 mM ammonium formate and 0.1% formic acid. Mycotoxins were eluted following a gradient elution program as follows: 0 min 1% B, 4.5 min 70% B, 5.5 min 90% B, 6 min 1% B and 7.0 min 1% B. The total chromatographic runtime was 7 min, with sample injection volume set at 3 µL, and a mobile phase flow rate of 0.7 mL min^−1^.

The mass spectrometry was operated in ESI+ mode, and data acquisition was carried out in scheduled multiple reaction monitoring (sMRM) mode. The capillary voltage and source temperatures were set at 4500 and 600 °C, respectively. Collision gas, ion source gas (GS1), ion source gas (GS2), and curtain gas were set at 9, 60, 60, and 35 psi, respectively. Two MRM characteristic transitions (1 precursor ion, 2 product ions) were monitored for each analyte. The selected MRM transitions and their respective analyte-dependent operating conditions, i.e., declustering potential (DP), collision cell exit potential (CXP), and collision energy (CE), are outlined in Table 2. Analyst^®^ Software 1.7.1 and SCIEX OS-Q Software were used for acquisition and processing of data.

### 2.6. Method Validation

The optimized LC-MS/MS method for the analysis of DON, ZEN, T-2, and HT-2 in oats was validated based on the acceptable performance criteria of analytical methods recommended by the European Commission regulations No. 2002/657/EC [28]. The performance characteristics evaluated were linearity, limit of detection (LOD), limit of quantification (LOQ), selectivity, matrix effect, recovery, repeatability, and reproducibility.

**Figure 1:**
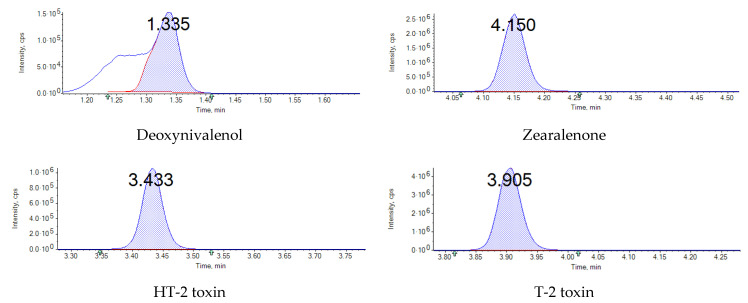
MRM chromatogram of investigated mycotoxins in mycotoxin-free oat samples spiked at 10 µg∙kg^−1^.

Linearity was evaluated using mycotoxin-free oat samples fortified with multi-mycotoxin working solutions at seven concentration levels (5–2000 µg∙kg^−1^ for T-2 and HT-2; 1–1000 µg∙kg^−1^ for DON and ZEN). Matrix effect or signal enhancement/suppression (SSE) was assessed by comparing peak areas of the matrix-matched standards spiked post-extraction with the corresponding peak areas of standards in pure solvent at the same concentration level. SSE (%) was calculated based on the slopes of calibration graphs obtained for each mycotoxin in neat standard solutions and spiked blank sample extracts using the formula: SSE (%) = 100 − (100 × [slope of spiked blank sample extract]/[slope of neat standard solution]). Recovery of each analyte was calculated as the peak area ratio of blank sample spiked before and after sample extraction, expressed as a percentage. The sensitivity of the developed method was determined by LODs and LOQs. LOD was defined as the concentration of each analyte that gave a peak with a signal-to-noise ratio (S/N) of 3, while LOQ was defined as the concentration of the analyte in spiked matrix giving S/N ratio of 10. Precision was determined by intra-day precision (repeatability) and inter-day precision (reproducibility). Intra-day precision was carried out by analysis of three replicates on the same day at three different concentration levels (10, 200, 1000 ppb) while inter-day precision was assessed by repeating the same procedure over three consecutive days. The data were used to calculate within-laboratory accuracy and precision and expressed as relative standard deviation (RSD). MRM chromatogram of investigated mycotoxins in spiked oat samples at 10 µg∙kg^−1^ is shown in Figure 1. The criteria for confirming a positive sample include a retention time within ±0.5% compared with the analyte in pure solvent, both qualifier and the quantifier with transitions above S/N ratio of 10:1, and the ion ratio of the quantifier and the qualifier transition within ±30%.

### 2.7. Statistical Analyses

The software R 4.0.5 (R Foundation for Statistical Computing, Vienna, Austria) together with the ggpubr (v0.4.0), faraway (v1.0.7), chisq.posthoc.test (v0.1.2), and MASS packages were used for statistical analyses [29,30,31,32,33]. All continuous variables were tested for normality of distribution by the Shapiro–Wilk test, and the null hypotheses were all rejected (*p* < 0.05). Frequency distributions (i.e., prevalence) were compared between groups by Pearson’s chi-squared test. Logistic regression was used to evaluate the effect of a continuous variable on a dependent, categorical variable, Spearman’s rank correlation was used to compare pairs of continuous variables, and the Kruskal–Wallis rank sum test was used to compare categories of a continuous variable. Where significant differences were identified in a group of categorical variables, post hoc pairwise comparisons were performed by Dunn’s rank-sum test using the Benjamini–Hochberg procedure for correcting family-wise error rate. Null hypotheses were rejected, and association considered significant at (*) *p* < 0.05; (**) *p* < 0.01; (***) *p* < 0.001. Finally, where pairwise significant differences were identified, post hoc power calculations were then also performed to ensure the reliability of the observation. Multiple logistic regression was used to model occurrence of each mycotoxin, and multiple linear regression was used to model mycotoxin levels amongst positive detections. Multivariate models were evaluated by Akaike Information Criterion to optimize selection of independent variables.

## 3. Results

### 3.1. Method Performance

The developed UPLC–MS/MS method is intended to be routinely employed for the quantitation of DON, ZEN, T-2, and HT-2 toxins in oats. Matrix-matched calibration was used to compensate for matrix effects and to ensure accurate quantification. The coefficients of determination (R^2^) for the tested mycotoxins were >0.999, showing a good linearity of the calibration curves. The LODs and LOQs for the target mycotoxins listed in Table 3 ranged between 0.9–2 µg∙kg^−1^ and 5–10 µg∙kg^−1^, respectively. Table 4 outlines the average recovery, repeatability (RSDr), and reproducibility (RSDR) values obtained from mycotoxin-free oat samples spiked at different concentration levels (10, 200, 1000 µg∙kg^−1^). The average recovery values ranged from 87% and 97%, which are considered satisfactory and similar to those obtained in other reports for cereals and cereal-based products. The RSDr and RSDR values of recoveries were below 3% and 7%, respectively, for the target mycotoxins. Both the recovery and RSD values were in accordance with the acceptable limits laid down by European Regulations [28], thereby confirming the adequate accuracy and precision of the current method.

### 3.2. Application of the Proposed Method to Real Samples

The proposed LC-MS/MS method was applied on real samples to quantitatively determine the prevalence of DON, ZEN, T-2, and HT-2 toxins in 202 oat samples. A total of 64 (32%) samples did not contain detectable levels of these four mycotoxins. Amongst the positive detections, type A trichothecenes (62%) were the most frequent contaminants, with T-2 and HT-2 each observed in 124 (61%) and 111 (55%) samples, respectively, and co-occurring in 109 (54%) samples. ZEN and DON were detected in 20 (10%) and 13 (6%) samples, respectively.

**Figure 2:**
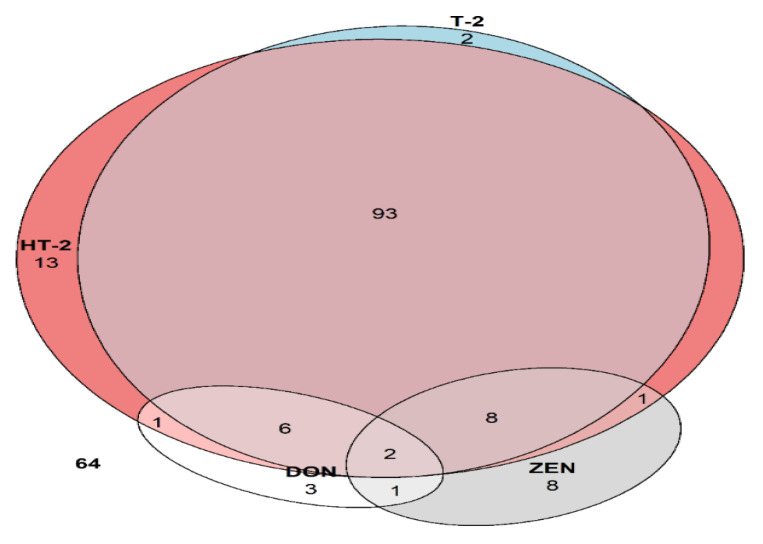
Euler diagram of deoxynivalenol (DON), HT-2 toxin (HT-2), T-2 toxin (T-2), and zearalenone (ZEN) detections.

As depicted in Figure 2, samples where either of the two type A trichothecenes was detected included the majority of DON (69%) and ZEN (55%) detections, though all four mycotoxins were only found in two (1%) samples. Only one mycotoxin was detectable in 26 (13%) samples, with half of those being HT-2. The most common pattern of occurrence was contamination with both type A trichothecenes only, which accounted for 67% of detected positive samples and 46% of all the samples analysed.

#### 3.2.1. Crop Season

The winter crop was represented by 99 (49%) samples and the spring crop by 103 (51%) samples. The prevalence of type A trichothecenes was significantly (*p* = 0.024) higher (by approximately 15%) in winter than in spring crops. The average T-2 level was double and the average HT-2 level was 20% higher than those in the spring crop. However, the type B DON (*p* = 0.026) and ZEN (*p* = 0.012) significantly decreased from 11% and 15% prevalence in winter to 2% and 4% in spring, respectively. The average spring crop ZEN concentration was double, while DON increased by 32%, compared to the winter crop average concentration.

#### 3.2.2. Harvest Date

The date of harvest was recorded for a total of 144 samples. Though the winter and spring, crops were harvested over periods of eight and seven weeks, respectively; most samples were obtained during the fourth week of harvesting each seasonal crop. Very weak correlations with harvest date were observed for the detected levels of DON (*r* = 0.35) and ZEN (*r* = 0.12), though neither relationship was considered significant (*p* > 0.05). Levels of Type A trichothecenes did not appear to be associated with the harvest date.

#### 3.2.3. County

**Figure 3:**
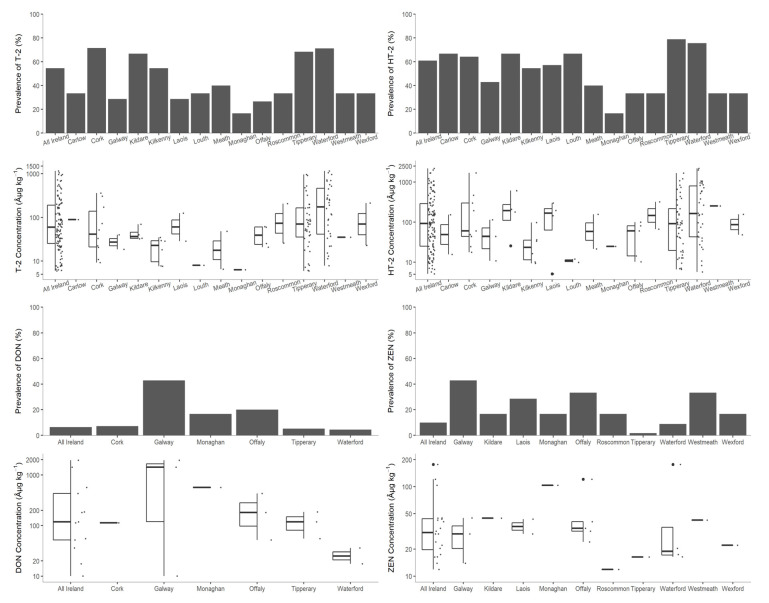
Prevalence and concentrations of deoxynivalenol (DON), HT-2 toxin (HT-2), T-2 toxin (T-2), and zearalenone (ZEN) by county.

The majority of samples were harvested from either Tipperary (*n* = 57) or Waterford (*n* = 45), followed by Offaly (*n* = 1 5), Cork (*n* = 14), and Kilkenny (*n* = 11). A further 14 counties were represented by less than ten samples each. The percentages of positive samples and the levels detected are illustrated in Figure 3.

Type A trichothecenes appeared to be more prevalent in Tipperary (79%), Waterford (76%), and Cork (71%), and prevalence exceeded 50% in another five counties, though only the frequency of occurrence in Tipperary (*p* = 0.035) was found to be statistically significantly different by post hoc analyses. Monaghan (17%) had the lowest prevalence, though the county was represented by only six samples. Conversely, of the six counties where DON was detected, Cork (7%), Tipperary (5%), and Waterford (4%) had the lowest prevalence, below Galway (43%), Offaly (20%), and Monaghan (17%). Among ten counties where ZEN was detected, Tipperary (2%) and Waterford (9%) had the lowest prevalence, while Galway (43%) had the highest. No statistically significant differences were observed amongst the detected levels of any mycotoxins in each county.

#### 3.2.4. Farming System

**Figure 4:**
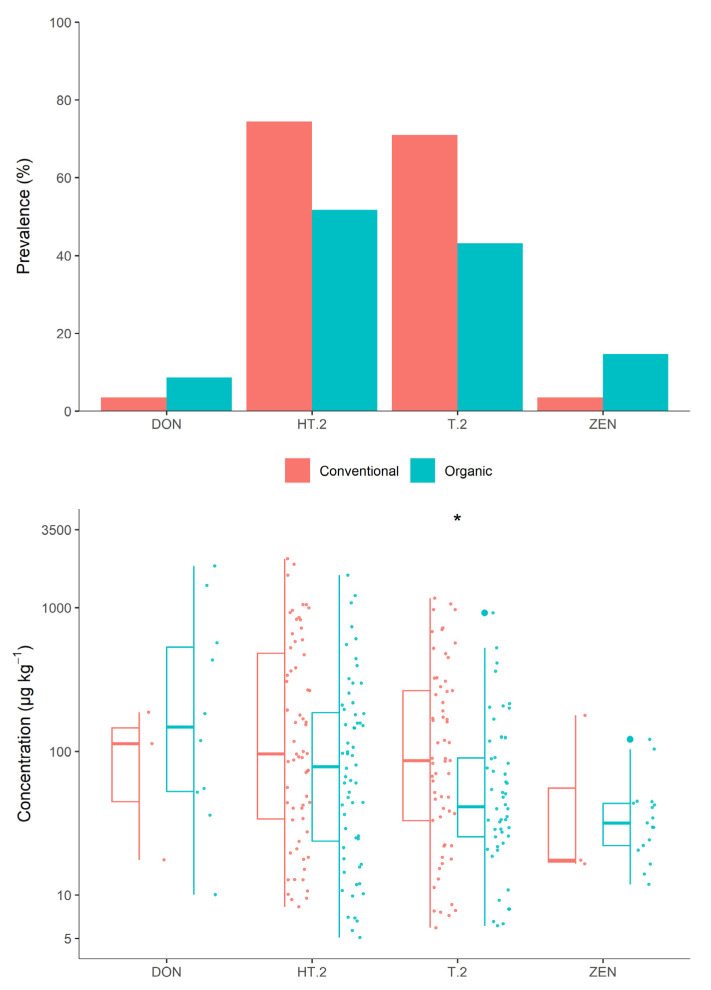
Prevalence of each mycotoxin (**top**) and the distribution of detected levels (**bottom**), by type of farming system.

There were 86 crops grown by conventional means, with fungicides applied, while 114 crops were grown using organic practices, and fungicide was not applied. Two crops were ambiguously recorded as organic, despite having fungicides applied, and were excluded from this comparison of farming systems. The mycotoxin detections among these samples are illustrated in Figure 4. The contamination with type A trichothecenes was significantly (*p* < 0.001) more prevalent in conventional (76%) crops compared to organic (53%). T-2 and HT-2 were each detected in 74% and 71% of conventional crops, but only 52% and 44% of organic crops, respectively. Again, DON and ZEN were found in a contrasting pattern, contaminating 9% and 15% of organic crops, respectively, but only 3% of conventional crops contained detectable levels of either mycotoxin.

The levels at which these mycotoxins were detected were not found to be significantly different between farming systems, with the sole exception of T-2. Both the mean and median concentrations of T-2 in conventionally grown crops were more than double those found in organic crops, and the overall distributions were found to be significantly different (*p* = 0.028). Though the mean HT-2 concentration was also 75% higher in conventional crops, the difference in distributions was not statistically significant, nor was the difference between farming systems for combined type A trichothecenes.

#### 3.2.5. Previous Crop

**Figure 5:**
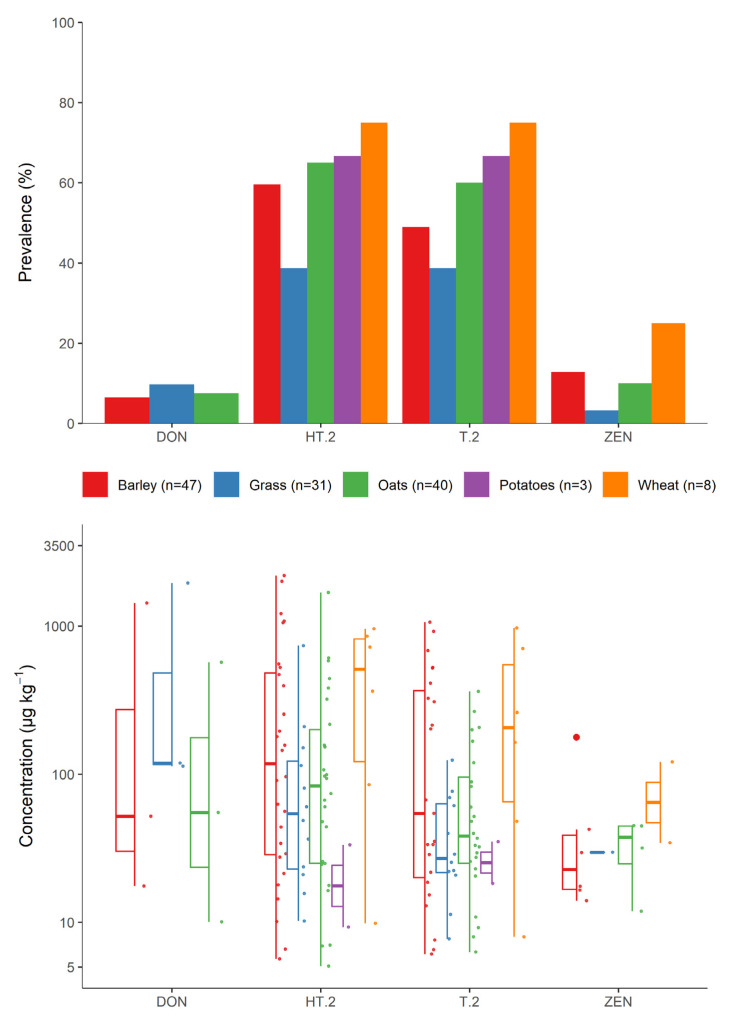
Prevalence of each mycotoxin (**top**) and the distribution of detected levels (**bottom**), by type of previous crop.

From 144 crops, the types of crop previously grown in the same field and their representation in the sample set are illustrated in Figure 5. Among these, barley (*n* = 47), oats (*n* = 40), and grass (*n* = 31) were the most common. The remaining 26 crops were split between 14 different combinations of previous crops and were excluded from this analysis. Oat crops grown after previous oat crops were found to have higher prevalence of all four mycotoxins, while oats grown after grass were lowest in mycotoxin prevalence, except for DON, which was lowest when grown after barley. Compared to oat crops grown after grass, type A trichothecenes were 1.8× more prevalent after barley crops, and 2.7× more prevalent after oats. The type of previous crop had a smaller effect on DON, which was found to have prevalence of 6%, 8%, and 10% in oat crops grown after barley, grass, and oats, respectively. ZEN was found in 13% of crops grown after oats or barley, but only 3% of crops after grass. However, none of the mycotoxin levels detected in the crops from each type of previous crop were found to be significantly different.

#### 3.2.6. Moisture

Except for one sample for which the moisture content was not recorded, the remaining 201 samples ranged between 11.0% and 28.6%, with a mean of 18.9% moisture. Around 57% of the recorded moisture levels were non-unique (i.e., representing more than one sample), therefore Spearman’s *r_S_* was the indicated test metric. However, no significant associations were identified between moisture levels and levels of any mycotoxin. On the other hand, logistic regression identified significant, very weak inverse relationships between moisture and the occurrence of both T-2 (*p* = 0.04) and HT-2 (*p* = 0.04).

#### 3.2.7. Oat Variety

The majority of the 172 crops, for which variety data were recorded, were represented by Husky (69%) oats. Isabel (11%), Barra (7%), Concert (3%), and Firth (2%) oats were also represented by multiple crops, while Delfin, Elyanne, and Sonas oats each accounted for one respective sample. A further 11 crops contained a mixture of two or more varieties and were considered a contiguous group (“Mixed”) for statistical analyses. However, no statistically significant differences were observed between the oat varieties, regarding either occurrence frequency or detected levels.

### 3.3. Multivariate Analysis

Initial models included all available independent variables: county, harvest week, moisture content, oat variety, previous crop, and season. The best fitting models were individually identified for the detected levels and the occurrence of each mycotoxin.

#### 3.3.1. T-2 Toxin

The occurrence of T-2 was best modelled by the type of farming system. Organic crops had a coefficient of −0.203 (*p* = 0.0498), while the test weight coefficient was 0.032 (*p* = 0.019) in this model. The levels of T-2 were best modelled by the previous crop, farming system, variety, and season. However, the only significant factors were previous crops of either grass (−256; *p* = 0.009) or oats (−168; *p* = 0.034).

#### 3.3.2. HT-2 Toxin

The best fitting model for the occurrence of HT-2 included only test weight, with a coefficient of 0.038 (*p* = 0.005). The levels of HT-2 were best modelled by farming system and variety. The model coefficients of the significant factors were the Sonas variety (1376; *p* = 0.0032) and the organic farming system (−353; *p* = 0.002).

#### 3.3.3. Type A Trichothecenes

Grouping T-2 and HT-2 together, the occurrence of these type A trichothecenes was best modelled by the type of farming system. The combined levels were best modelled by previous crop, type of farming system, and variety. The model coefficients of significant factors were grass (−701; *p* = 0.0036) or oats (−378; *p* = 0.046) as the previous crop, an organic farming system (−469; *p* = 0.012), or the Sonas variety (1604; *p* = 0.019).

#### 3.3.4. Deoxynivalenol

The best fitting model for the occurrence of DON included only moisture, though the association was not significant (*p* > 0.05). Levels of DON were best modelled by the previous crop and variety. The model coefficients of the significant factors were mixed oat varieties (−3228; *p* = 0.0093), Husky variety (−1395; *p* = 0.029) and grass as the previous crop (1913; *p* = 0.012).

#### 3.3.5. Zearalenone

The best fitting model for the occurrence of ZEN included the season and previous crop, though only the Combi Crop category of the previous crop was considered significant, with a model coefficient of 0.228 (*p* = 0.036). Quantitative levels of ZEN were best modelled by the variety and type of farming system. However, none of the factors were calculated to have statistically significant effects on the model.

## 4. Discussion

More than 300 species of *Fusarium* were reported to cause economically important diseases, such as *Fusarium* head blight of wheat and barley, as well as ear rot of maize and panicle blight of oats, resulting in huge decreases in both the yield and quality of crops [4,5]. Aside from the poor crop yield and quality, *Fusarium* species also produce a vast number of mycotoxins in infected grains. The most toxicologically important *Fusarium* mycotoxins are trichothecenes (including DON, T-2, and HT-2 toxins), ZEN and fumonisin B1 [4,6,34]. Other notable “emerging” *Fusarium* mycotoxins include moniliformin, nivalenol, and diacetoxyscirpenol [4]. In the current study, 202 unprocessed Irish oats samples collected between July and December 2020, from different agricultural systems, were analysed for the occurrence of major *Fusarium* mycotoxins—DON, ZEN, T-2 and HT-2. Furthermore, we evaluated the impact of certain agronomic practices before or during oat production on the levels of DON, ZEN, T-2, and HT-2. Of the four mycotoxins investigated, T-2 and HT-2 were the most frequently detected. Approximately 55% and 61% of analysed samples were positive for T-2 and HT-2, at levels ranging between 5.9–1165 µg∙kg^−1^ and 5.1–2190 µg∙kg^−1^, respectively. The mean levels for T-2 and HT-2 were 163.6 µg∙kg^−1^ and 264.0 µg∙kg^−1^, respectively. 

Currently, there are no legal maximum limits for these toxins; however, the EC has published an indicative level of 1000 µg∙kg^−1^ for the sum of T-2 and HT-2 (HT-2 + T-2) in unprocessed oats [35]. Moreover, due to the large year-to-year variation in the occurrence of T-2 and HT-2 toxins, the EC recommended the collection of more data on T-2 and HT-2 in cereals and cereal products and the identification of various factors promoting the relative high levels of T-2 and HT-2 toxins in cereals [35]. In this study, 15 samples (7.4%) were found to contain levels of combined T-2 and HT-2 above the EU recommended limits (Table 5). The previous mycotoxins surveys also showed that oats produced particularly in Northern and Western Europe were more susceptible to T-2 and HT-2 contamination compared to other cereals such as wheat, barley, and rye [15,36,37,38]. A very high prevalence of T-2 and HT-2 toxins was observed in 458 unprocessed oat samples, collected between 2002 and 2005, from various oat fields in the UK [36]. T-2 and HT-2 toxins were detected in 84% and 92% of the samples analysed, with combined mean levels (HT-2 + T-2) of 570 µg∙kg^−1^ and a maximum concentration of 9990 µg∙kg^−1^. Similarly, Edwards et al. [37] reported a mean of 450 µg∙kg^−1^ for the total levels of T-2 and HT-2 in UK oats samples collected over three seasons (2006 to 2008). Unprocessed oats samples (representative of Irish oat production in 2015 and 2016), analysed for multi-mycotoxins, showed a very high prevalence of T-2 and HT-2 toxins (51%), with a mean concentration of 770 µg∙kg^−1^ [15]. High levels of T-2 and HT-2 were also detected in oats from Norway [38,39], Finland [40], Sweden [41], and Switzerland [42].

Many studies showed that *F. langsethiae* and closely related *Fusarium* species including *F. sporotrichioides*, *F. poae*, *F. sibiricum* and *F. armeniacum* are the common *Fusarium* species associated with oat infection and the production of T-2 and HT-2 [42,43,44]. However, in most of Europe, *F. langsethiae* and *F. poae* appear to be the predominant T-2 and HT-2 producers, as they are frequently detected and isolated from infected cereals [42,44,45]. Moreover, both species occurred in the high levels of T-2 and HT-2 toxins, mainly in oats [36,37]. An analysis of 240 UK oat samples with known concentration of T-2 and HT-2 toxins showed that *F. langsethiae* was prevalent in 100% of the samples. A strong positive correlation (*p* < 0.001, r^2^ = 0.60) was also observed between *F. langsethiae* DNA and the T-2/HT-2 content of oats [45]. Similar results were also reported for oats in other European countries, including Italy and Switzerland [42,46]. For reasons that remain elusive, oat crops are more susceptible to strains of *F. langsethiae*, compared to other cereals such as wheat and barley [36,37,47]. However, as they are less aggressive (reduced pathogenicity) compared to other *Fusarium* pathogens [48], they do not produce any visible symptoms in crops; they are mostly isolated from symptomless oat crops [44,48]. Therefore, innovative tools for the early and rapid detection of *F. langsethiae* and other T-2- and HT-2-producing *Fusarium* species are needed for effective interventions, and to reduce the levels of T-2 and HT-2 in oats intended for human consumption. 

In contrast to T-2 and HT-2, the incidences and concentrations of DON and ZEN in the samples analysed were generally low (Table 5). This implied that the environmental requirements of T-2/HT-2-producing *Fusarium* species were different from *F. culmorum* and *F. graminearum*, the major producers of DON and ZEN. De Colli et al. [15] and Edwards et al. [36,37] also detected low levels of DON and ZEN in Irish and UK oat samples, respectively. However, higher concentrations of DON and ZEN were detected in Swedish and Norwegian oat samples [41,42]. This may reflect different agroclimatic factors and predominant mycotoxigenic fungal species.

Several agronomic factors including crop rotation, sowing date, fungicide treatment, and tillage [49,50], were shown to significantly affect the severity of *Fusarium* head blight and the contamination of cereals with *Fusarium* mycotoxins. In this study, seven agronomic fixed factors (crop season, harvest date, county, farming system, previous crop, moisture, and oat variety) were studied in relation to the concentrations of four major *Fusarium* mycotoxins in Irish oat crops—DON, ZEN, T-2 and HT-2. Overall, the previous crops, farming system, and crop season were the main factors identified as significantly influencing the mycotoxin content of the analysed Irish oat samples. The other factors, harvest date, county, moisture, and oat variety, had little significant effect.

### 4.1. Crop Season

In this study, the concentrations of T-2 and HT-2 were significantly higher in winter sown oat samples compared with spring sown oat samples, while the DON and ZEN contents of spring oat samples were higher than winter oats. This is consistent with field surveys carried out by Edwards et al. [36,37], Hietaniemi et al. [39], Hofgaard et al. [40], Nathanail et al. [51], and Opoku et al. [47], who reported significantly higher levels of T-2 and HT-2 in winter oats compared to spring oats. However, as our data were highly skewed with many poorly represented varieties, it was difficult to statistically determine if the results observed were due to varietal differences or climatic conditions. Under controlled experimental conditions, Imathiu et al. [44], examined the effect of oat varieties on *Fusarium* infection and the mycotoxin content of cereals, including oats and wheat. They observed higher *F. langsethiae* DNA and T-2/HT-2 contents in winter oat varieties compared to spring oat varieties [44]. These authors suggested that the genetic differences between the oat varieties may be responsible for higher concentrations of T-2 and HT-2 toxins found in winter oats. Additionally, as winter oat heads emerge earlier than spring oats, this may predispose panicles of winter sown oats to the extended *F. langsethiae* infection, resulting in the accumulation of more T-2 and HT-2 compared to spring oats [44,48]. Contrary to our findings, Irish spring oats samples collected between 2015 and 2016 were found to contain higher levels of T-2 and HT-2 compared to winter oats [15]. Additionally, a three-year survey of French barley found higher levels of T-2 and HT-2 in spring-compared to winter-sown barley [52]. Field experiments under similar agroclimatic factors across several years and locations are needed to fully understand and establish the influence of oat varieties on mycotoxin contamination.

### 4.2. Previous Crop

*Fusarium* head blight epidemics and elevated mycotoxin concentrations are generally considered to originate from inoculum associated with stubble and straw, particularly from small-grain cereals [53]. Many researchers showed that the cultivation of cereals, particularly maize, increases the risk of *Fusarium* head blight and mycotoxin contamination in subsequent oats, wheat, and barley crops, compared with other previous crops, especially non-cereal crops [36,37,38,39,40]. In this study, oat crops grown after previous oat crops were found to have higher prevalence of all four mycotoxins, while oats grown after grass and non-cereal crops were lowest in mycotoxin prevalence. Moreover, we observed that T-2 and HT-2 were 2.7× more prevalent when grown after oats and 1.8× more prevalent after barley crops. This indicates that inoculum is largely present in oat crop debris carried into the next crop season and could explain the higher prevalence of mycotoxins observed in this study. A higher contamination of oats with T-2 and HT-2 toxins from fields, with cereals as the previous crop, was also observed by Edwards et al. [36,37] and Bernhoft et al. [49,50]. A similar study on *Fusarium* mycotoxins in Switzerland also showed that growing maize prior to barley resulted in higher *F. graminearum* incidences and mycotoxin contents in barley grains, compared with other preceding crops such as canola or potatoes [41]. Previous crops, especially cereals, may leave a large number of infected residues in the field, which are then carried over into the next cropping season. These advanced cultures may lead to the contamination of new crops with an increased level of mycotoxins, particularly in susceptible varieties. Orlando et al. [52], Bernhoft et al. [50], and Edwards et al., [37] observed a significantly lower T-2 and HT2 level in oat crops that followed non-cereal crops. Thus, the implementation of crop rotations with non-host plant species such as canola, pea, potato, and sugar beet plants, and practices such as crop debris management and ploughing, could help prevent or minimise *Fusarium* infection and mycotoxin contamination of cereal crops.

### 4.3. Farming System

Due to the concerns about conventional agricultural practices, including environmental impact and food safety, the interest in organic products has increased worldwide [54]. Generally, organic farming practices make use of smaller fields, more crop rotation, and no use of pesticides or mineral fertilisers [54]. However, factors including elevated moisture conditions known to promote fungal growth and mycotoxin production in cereals are often associated with organic farming practices. Therefore, several researchers attempted to investigate and compare the mycotoxins content of organic crops. Most studies on this topic, including a report by FAO, found either no significant differences between the mycotoxin content of organically and conventionally grown cereals or a higher concentration of mycotoxins in organic samples compared to conventional samples [19,55,56,57,58]. However, a few other researchers reported that the implementation of organic practices significantly reduced *Fusarium* infection and mycotoxin contamination [36,37,50]. For instance, up to 602 organic cereals samples collected at harvest, between 2002 and 2004, in Norway contained significantly lower *Fusarium* infestation and concentrations of important mycotoxins—DON, ZEN, T-2, and HT-2—compared with conventional cereal samples. Furthermore, the levels of T-2 and HT-2 detected in organic oats were lower compared with conventional oat samples. Similarly, analyses of organic and conventional oat and oat-based products in Germany and Norway showed low incidences of HT-2 and T-2 in organic samples compared to conventional samples [50]. Additionally, concentrations of T-2 and HT-2 in cereal samples collected from different farms across UK were five times higher in conventionally grown cereals samples compared to samples from organic farms [36,37]. In this study, the lower prevalence of mycotoxins, particularly T-2 and HT-2, in organic oat samples might be explained by less intensive tillage/ploughed soils and by the longer crop rotations that are more diverse and less cereal intensive. These are primary factors which differentiate organic farming practices from conventional practices and were shown to not only reduce the severity of *Fusarium* infection, but also minimise mycotoxin contamination, in spite of eschewing fungicide use [36,37,53].

## 5. Conclusions

In this study, seven agronomic fixed factors (county, crop season, farming system, harvest date, moisture, oat variety, previous crop, and test weight) were investigated for association to the occurrence of four major *Fusarium* mycotoxins in Irish oat crops. Though mycotoxins were detectable in the majority of crops, less than a tenth of all crops assayed were found to exceed the European regulatory limits. Most commonly, both type A trichothecenes were present, while DON and ZEN occurrences were relatively infrequent. The type of farming system and species of crop previously grown in the same field were the main factors identified to significantly influence mycotoxin prevalence and concentration. Particularly, the contamination by type A trichothecenes was observed to decrease when adhering to an organic farming system, or when growing the sampled oats after a previous crop of grass. Other factors, such as county, harvest date, moisture, and oat variety were less significant, though some factors were underrepresented in this dataset. Particularly, the different growing varieties of oat crops, and their growing after different species of cereal and non-cereal previous crops, may be considered as particularly interesting variables to help structure future studies.

## Figures and Tables

**Table 1:** Maximum EU limits for deoxynivalenol, zearalenone, and the sum of T2 and HT-2 toxins in unprocessed cereals and finished products intended for human consumption. For T-2 and HT-2 toxins, no limit values are defined, but only indicative values.

Mycotoxin	Food	Maximum Level (µg∙kg^−1^)
Deoxynivalenol	Unprocessed oats.	1750
	Oat flour, meal, bran, or germ.	750
	Bread, pastries, biscuits, cereal snacks, and breakfast cereals.	500
Zearalenone	Unprocessed oats.	100
	Oat flour, meal, bran, or germ.	75
	Bread, pastries, biscuits, cereal snacks, and breakfast cereals.	50
T-2 and HT-2 toxin	Unprocessed oats.	1000
	Oat grains for direct human consumption.	200
	Oat bran and flakes.	200

**Table 2:** MS/MS parameters for the determination of DON, ZEN, T2, and HT2 in oats.

Mycotoxin	Molecular Formula	Molar Mass	Measured Ion	Precursor Ion (*m*/*z*)	Product Ion (*m*/*z*)	Retention Time	DP (V)	CE (V)	CXP (V)
Deoxynivalenol	C_15_H_20_O_6_	296.31	[M + H]^+^	297.1	249.1 203.2	1.34	91 91	21 21	20 20
Zearalenone	C_18_H_22_O_5_	318.364	[M + H]^+^	319.1	301.1 283.1	4.15	81 81	15 17	16 14
T-2 toxin	C_24_H_34_O_9_	466.52	[M + NH_4_]^+^	484.3	215.2 185.1	3.90	76 76	29 31	18 11
HT-2 toxin	C_22_H_32_O_8_	424.5	[M + NH_4_]^+^	442.2	263.1 215.1	3.43	71 71	19 19	14 22

DP—declustering potential, CE—collision energy, CXP—collision cell exit potential.

**Table 3:** Linearity data, limit of detection (LOD), limit of quantification (LOQ), and matrix effect (SSE) for tested compounds by LC-MS/MS.

Analyte	Calibration Range (µg-kg^−1^)	Graph Equation	Linearity R^2^	SSE (%)	LOD (µg∙kg^−1^)	LOQ (µg∙kg^−1^)
Deoxynivalenol	10–2000	y = 594.7x + 372.25	0.9994	−10	0.9	10
Zearalenone	10–2000	y = 15,828.4x + 3148.16	0.9992	−7.8	2	10
T-2 toxin	5–2000	y = 46,303x + 5172.73	0.9996	6.8	1.5	5
HT-2 toxin	5–2000	y = 39,794x + 102.53	0.9998	−9.3	1	5

**Table 4:** Results of recovery and precision of the target mycotoxins in the analysed oats (n = 3).

	10 µg∙kg^−1^			200 µg∙kg^−1^			1000 µg∙kg^−1^		
	Recovery (%)	RSDr ^b^ (%)	RSDR ^c^ (%)	Recovery (%)	RSDr ^b^ (%)	RSDR ^c^ (%)	Recovery (%)	RSDr ^b^ (%)	RSDR ^c^ (%)
Deoxynivalenol	87	2.6	4.6	91	2.5	4.8	91	1.9	3.1
Zearalenone	92	3.1	3.9	90	2.9	3.1	96	2.2	6.2
T-2 toxin	91	2.5	5.2	92	2.1	4.6	95	1.7	4.3
HT-2 toxin	93	1.1	5.4	92	1.9	4.8	96	2.1	4.6

^b^ RSDr for repeatability; ^c^ RSDR for reproducibility.

**Table 5:** Basic descriptive statistics for the detection of mycotoxins in oat samples, including samples over the European Commission’s regulatory limits in unprocessed oats: 1750 µg∙kg^−1^ deoxynivalenol; 100 µg∙kg^−1^ zearalenone; 1000 µg∙kg^−1^ for the total T-2 and HT-2 toxins, applied here to T-2 and HT-2 individually.

*N* = 202	Deoxynivalenol	Zearalenone	T-2 Toxin	HT-2 Toxin	T-2 + HT-2
Prevalence (%)	13 (6.4)	20 (9.9)	111 (55)	124 (61)	126 (62)
Exceeding Limit (%)	1 (0.5)	3 (1.5)	2 (1.0)	8 (4.0)	15 (7.4)
Median (µg∙kg^−1^)	119	31	61	93	138
Range (µg∙kg^−1^)	10–1947	12–177	6–1166	5–2190	5–3064
